# Hydrogen solution exposure at a seasonal timescale does not affect the geomechanical properties of clay-rich sandstones

**DOI:** 10.1038/s41598-025-19743-8

**Published:** 2025-10-14

**Authors:** Milad Naderloo, Hadi Hajibeygi, Anne Pluymakers

**Affiliations:** Department of Geoscience and Engineering, Delft University of Technology, Delft, the Netherlands

**Keywords:** Engineering, Solid Earth sciences

## Abstract

Underground hydrogen storage (UHS) in underground geological reservoirs is a promising solution for large-scale energy storage. However, several challenges, particularly geomechanical ones, must be resolved before UHS can be widely and safely deployed. The interactions between hydrogen, brine, and reservoir rock, combined with the cyclic stresses resulting from hydrogen injection and withdrawal may affect the mechanical integrity of the reservoir, the caprock, as well as its surrounding formations. This is an experimental investigation into the geomechanical impact of a 6 month exposure of clay-rich sandstone (Yellow Felser) rocks to hydrogen and/or brine. Cm-scale samples were exposed to hydrogen-saturated brine at 150 bar and $100^{\circ }\hbox {C}$ in an autoclave for the period of six months. Afterwards, triaxial cyclic loading experiments were conducted on the samples under confining pressures of 10, 20, and 30 MPa. The results are compared with those from the reference samples, which have been exposed to brine only, for the same time period. Each mechanical test included eight stress cycles in the linear stress regime (below the brittle yield point), followed by loading to failure. The frequency, amplitude, and stress conditions were tailored to each confining pressure. The results showed that six months of hydrogen-saturated brine exposure had no noticeable effect on the failure envelope, elastic properties, inelastic strain, and acoustic properties of the Yellow Felser sandstone compared to exposure to brine alone. Internal friction, P-wave velocity, and Young’s modulus each showed a change of around 3%, which is on the same order as the repeatability and therefore indicating minimal geomechanical alteration. Complementary qualitative and quantitative scanning electron microscopy (SEM) analyses revealed negligible microstructural changes. When eight stress cycles were applied within the linear stress regime, the majority of inelastic strain occurred during the first cycle, with no progressive accumulation thereafter. A comparison with samples tested under monotonic loading to failure confirmed that cyclic loading under these conditions does not affect the rock strength of Yellow Felser sandstone. These findings provide new insights into the combined effects of cyclic stress and hydrogen/brine/rock interactions on the geomechanical behavior of clay-rich sandstones under reservoir-relevant pressure and temperature conditions.

## Introduction

The growing urgency of climate change has intensified the global demand for clean and renewable energy sources. Technologies such as hydropower, geothermal, wind, and solar energy are essential to achieving net-zero carbon emissions worldwide^1^. By 2050, renewable energy is projected to fulfill 45% of the global energy demand, in line with the International Energy Agency (IEA)’s goal of reaching net-zero emissions^2^. Reliable supply of renewable energy depends on balancing supply and demand, which are influenced by unpredictable intermittent weather conditions. Surplus energy generated from solar and wind sources can be converted into hydrogen and stored in large (TWh) scale. This conversion can be accomplished through various methods, including the production of “green hydrogen”. This term refers to hydrogen produced via water electrolysis using surplus renewable energy sources^3^. Storing renewable energy on a large scale across various locations can indeed mitigate the supply and demand imbalance^4^. Geological formations, including depleted reservoirs (with TWh-scale capacity) and salt caverns (each with GWh-scale capacity), have demonstrated their effectiveness as storage sites for energy-rich or energy-carrying fluids such as compressed air, heated water, and hydrogen^5,6^. Therefore, hydrogen storage in geological formations such as depleted gas reservoirs and aquifers is seen as an essential compnent of a viable energy transition, since it will ensure a stable energy supply^7,8^. Despite these advantages, however, the implementation of underground hydrogen storage (UHS) technology poses significant technical challenges, particularly concerning the geomechanical and mechanochemical integrity and stability of the storage reservoirs and their caprock seals^9,10^.

In the context of porous media, the direct interaction among hydrogen gas (H_2_), brine, and reservoir rock (minerals) as well as the subsequent contact of hydrogen after its dissolution in brine has the potential to initiate abiotic (geochemical) reactions^11–15^. The efficacy of abiotic reactions are contingent upon various operating conditions, including but not limited to temperature, pressure, salinity, pH, rock wettability, and rock-fluid interfacial tension^16,17^. Consequently, a pivotal challenge in UHS pertains to the comprehension of the interaction between hydrogen, brine, and reservoir rock, which has the potential to modify the geomechanical properties of the storage formation^9^. The aforementioned alterations have the potential to compromise the integrity of both the storage formation and the overlying caprock. This, in turn, poses a risk of leakage and loss of containment^18,19^. The abiotic interactions of hydrogen with reservoir and caprock minerals, i.e. excluding microbial activity, can lead to mineral dissolution (e.g., carbonate, sulfate, feldspar, clay) and precipitation (e.g., illite, sulfide, pyrrhotite)^11,20,21^. A growing body of experimental and modeling studies has investigated the geochemical reactivity of hydrogen with geological formations relevant to underground storage. Truche et al.^21,22^ conducted studies on the geochemical effects of hydrogen on clay-rich rock containing 1-2 wt% framboidal pyrite under temperatures of $90-250^{\circ }\hbox {C}$ and hydrogen partial pressures of 3-30 bar. Their results indicated that hydrogen destabilizes pyrite, leading to sulfide production, with rapid pyrite-to-pyrrhotite conversion occurring above $90^{\circ }\hbox {C}$ and 10 bar H_2_. In six-week static batch experiments under varying reservoir conditions (pressure, temperature, and salinity), sandstones were exposed to 100% hydrogen. Petrographic analysis showed no hydrogen interaction with Tertiary/Molasse sandstones from Austria. However, Permian (Altmark) and Triassic (Emsland) sandstones, containing carbonate and anhydrite cements, exhibited petrographic and petrophysical changes^12^. Hassanpouryouzband et al.^13^ conducted over 250 batch reaction experiments on various reservoir sandstones under subsurface-relevant conditions (332.15-353.15 K, 0.1-200 bar) for 2-8 weeks to assess hydrogen reactivity. The results confirm no risk of hydrogen loss or reservoir integrity degradation due to abiotic geochemical reactions in sandstone reservoirs. Al-Yaseri et al.^23^ exposed Bandera Gray Sandstone and Indiana Limestone to H_2_-CH_4_ mixture at $75^{\circ }\hbox {C}$ and 1400 psi for 90 days. Nuclear Magnetic Resonance (NMR) and gas porosity analysis revealed minimal reactivity and negligible changes in petrophysical properties. The geochemical interaction of hydrogen with sandstone was investigated through experiments and numerical modeling. Experiments were primarily conducted at $100^{\circ }\hbox {C}$, with some at $200^{\circ }\hbox {C}$, under hydrogen pressures up to 100 bar for durations of 1.5 to 6 months. Results showed minimal reactivity between sandstone minerals and hydrogen. Combined with numerical findings, the study suggests that injected hydrogen remains largely inert^18^. Geochemical modeling of hydrogen/brine/rock interactions in Polish Lowland sandstones, mudstones, and claystones revealed that pH strongly influences reaction extent, with goethite playing a key role in hydrogen consumption. Notably, porosity changes were much smaller in sandstones compared to mudstones and claystones^14^. Bensing et al.^24^ conducted batch-reactor experiments on calcite-bearing claystone under 15 MPa, at $\sim 25\, ^{\circ }$C, and in 10 wt% $\hbox {NaCl-SO}_{4}^{2-}$ brine for 30 days. These experiments revealed significant hydrogen-induced dissolution of calcite fossils, which initiated intragranular porosity. A synthesis of findings from recent literature reveals that most silicate phases–including quartz, feldspar, and kaolinite–consistently exhibit negligible reactivity under conditions relevant to UHS. In contrast, iron-bearing clays have demonstrated moderate redox activity when exposed to hydrogen, although the extent of these reactions is variable and generally limited. More notably, carbonate and sulphate minerals, e.g., calcite, dolomite, siderite, gypsum, and anhydrite–have been, are shown to undergo dissolution as exposed to hydrogen under UHS conditions, indicating a higher potential for their geochemical property alteration^13,18,23,25^.

The UHS literature is mainly focused on evaluation of hydrogen transport and trapping characteristics, petrophysical properties, and fluid composition analysis. Despite its crucial role in the safety of operations, the geomechanical effects of hydrogen exposure on reservoir rocks have not yet been fully investigated. More precisely, the influence of the aforementioned reactions on geomechanical properties of sandstone rocks remains to be elucidated. Mineral precipitation and dissolution have the capacity to modify porosity and mineral morphology. These alterations, in turn, have the potential to influence rock strength, elastic properties, and, consequently, the mechanical integrity of the reservoir and caprock^26^. A limited number of experimental studies have been conducted to explore the effects of prolonged exposure to hydrogen-saturated brine under representative subsurface conditions. These studies have quantified alteration of the rock strength, elastic properties, and the failure mechanisms^27,28^. A recent study has exposed dry sandstone, shale, and limestone cores to hydrogen at 3.45 MPa for different time duration of 30 days for shale and limestone, and 90 days for sandstone rocks^28^. The changes in petrophysical properties were exclusively observed for shale rocks, with as high as 50% increase in their porosity and a twofold increase in their permeability. In contrast, sandstone and limestone rocks exhibited negligible alterations of their properties. Furthermore, the presented geomechanical analysis revealed that sandstone rocks exhibited no observable property alteration, while limestone rocks demonstrated a substantial increase in their Young’s modulus, and shale samples exhibited a negligible reduction in their ultimate strength^28^. In another study, performed by Dabbaghi et al.^27^, sandstone samples from the Sundance Formation were treated with brine and varying hydrogen concentrations (50% and 100%) under 15 MPa and $83~^{\circ }$C for two weeks. Specimens exposed to hydrogen exhibited 24–41% lower peak strength compared to brine-only exposed samples. XRD analysis revealed a reduction in dolomite content of these rocks, indicating hydrogen-induced mineral dissolution and degradation of their mechanical and elastic properties. It is clear that the influence of hydrogen-saturated brine exposure on mechanical integrity of the sandstone rock samples remains as a knowledge gap in the literature. Therefore, there is a need for its systematic investigations to thoroughly assess how hydrogen-saturated brine interactions influence the mechanical behavior of various reservoir lithologies, and whether it alters their strength under cyclic loading.

Given that when geochemical alterations are found, they are found in clay-rich rocks and not in pure sandstones, as well as that the vast majority of experiments lasted shorter than two months, we added three modifications compared to current literature. First, in this study, the initial focus is on the geomechanical alterations occurring of a clay-rich sandstone (Yellow Felser). Second, it was not exposed to pure hydrogen but to a hydrogen-saturated brine, which is a better reflection of the expected geochemistry in-situ. Third, the samples were sitting in an autoclave for a duration of six months at a pressure of 150 bar and a temperature of $100^{\circ }\hbox {C}$, in an attempt to maximize the potential for geochemical reactions. Apart from the conventional geochemical analyses, this was followed by triaxial cyclic loading tests, to evaluate changes in strength, elastic properties, inelastic strain, and to estimate potential changes in the failure envelope. Subsequently, a qualitative and quantitative analysis was performed on SEM images of sandstone samples exposed either solely to brine or to a combination of hydrogen and brine. The objective of these analyses was to identify changes in pore and mineral structure. This research therefore offers novel and critical insights into the combined effects of cyclic stress behavior and potential hydrogen-induced abiotic reactions on the geomechanical properties relevant to UHS in clay-rich geological formations.

## Material and methods

### Sample preparation and and exposure apparatus

**Fig. 1:**
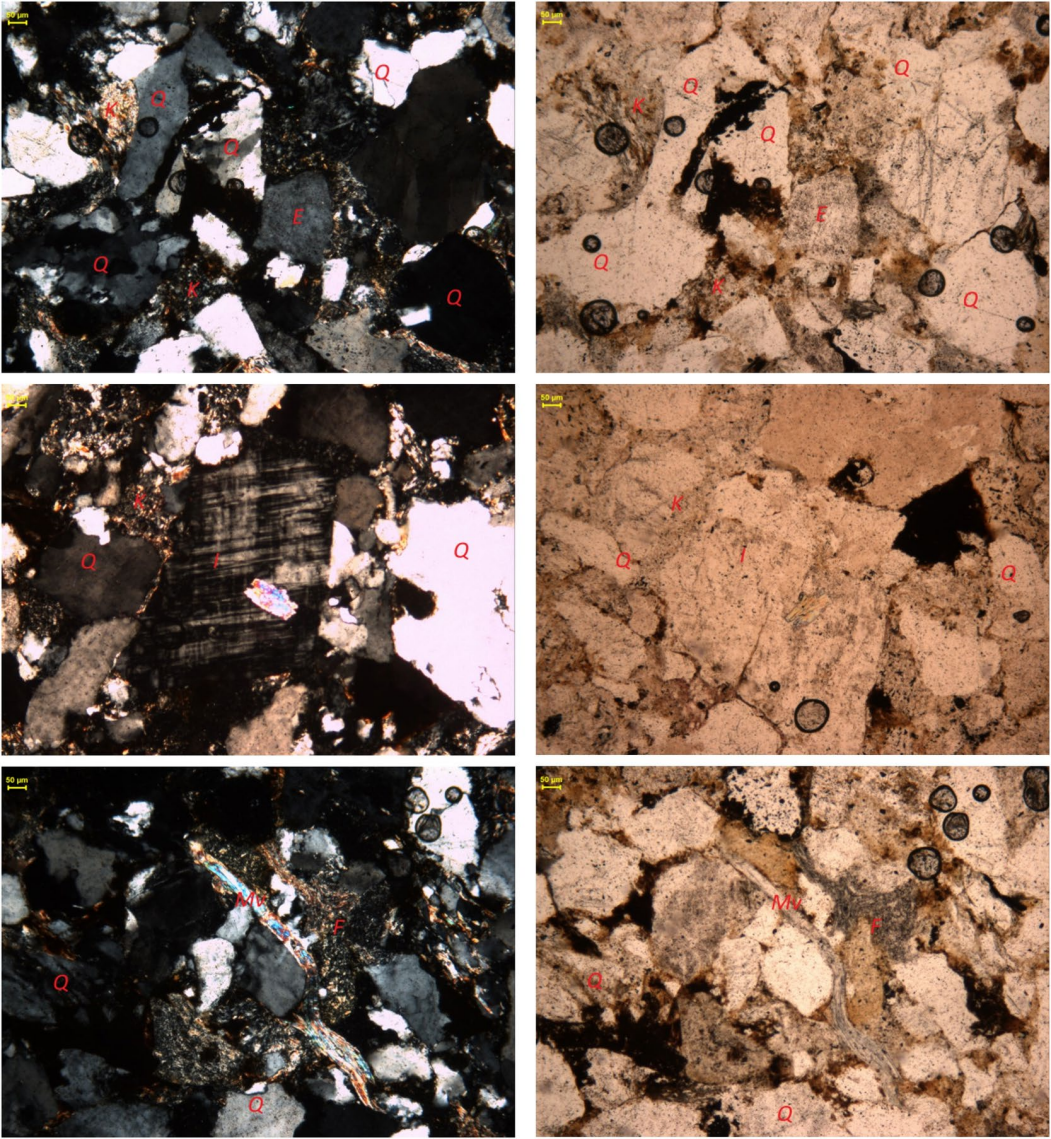
Thin section images of rock samples showing the distribution of various minerals and porosity, identified using the following symbols: Quartz (Q), Feldspar (F), Illite (I), Kaolinite (K), Muscovite (Mv), and Epidote (E), as referenced in the accompanying table. Images were acquired using a microscope with 10X magnification in the binoculars and 4-50X in the objective lenses. The left-hand side images were taken under polarized light, while the right-hand side images were captured under normal transmitted light. Each mineral or feature is labeled accordingly in the images^29^.

**Fig. 2:**
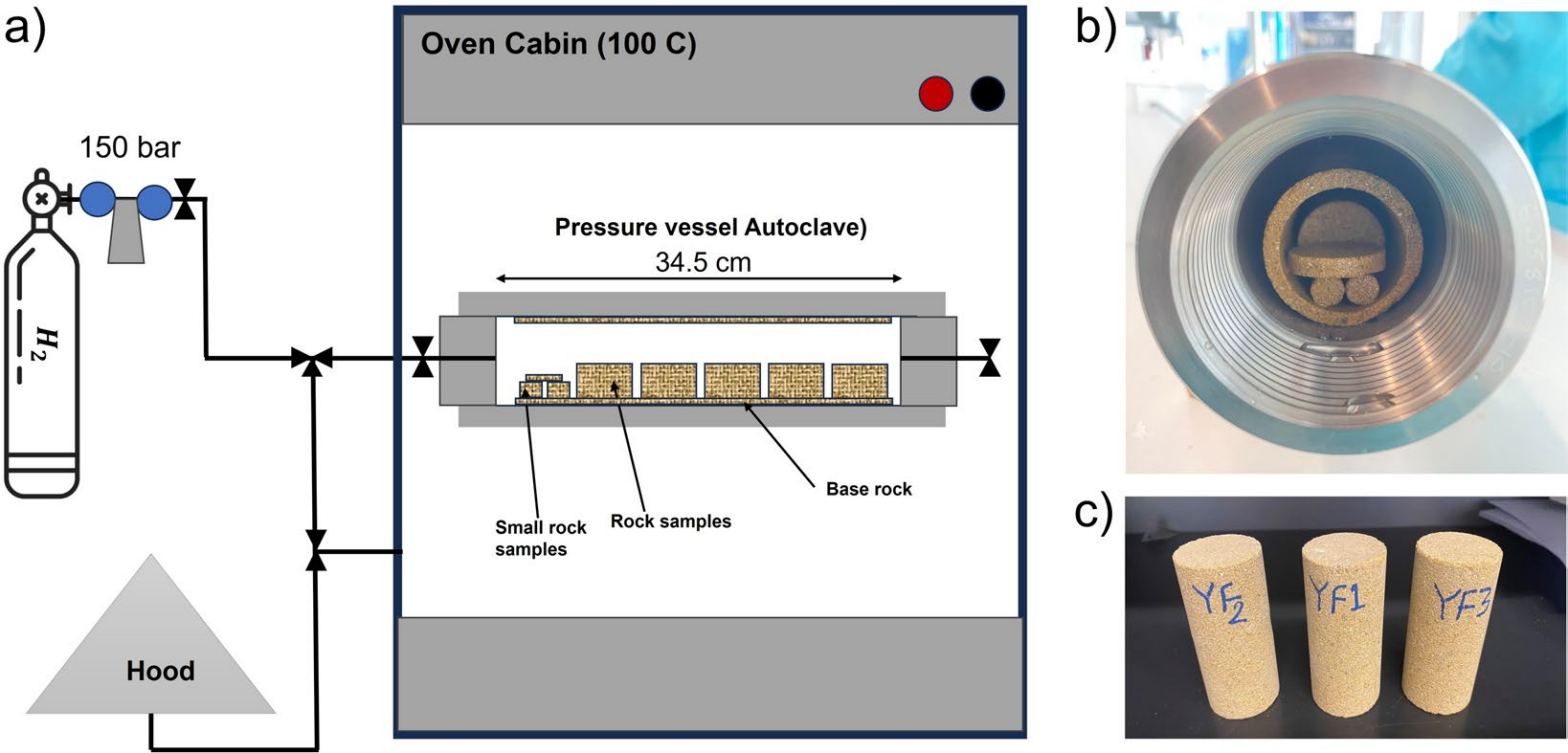
Experimental setup used for hydrogen/brine exposure experiments. (**a**) Schematic of the pressure vessel (autoclave) placed in an oven at $100^{\circ }\hbox {C}$, with connections for hydrogen gas supply and a fume hood. (**b**) Top view of the pressure vessel showing the arrangement of rock samples inside the autoclave. (c) Cylindrical Yellow Felser sandstone samples prepared for the exposure experiments.

Yellow Felser sandstone samples are used for exposure to hydrogen-saturated brine tests, as shown in Fig. 2c. The samples are homogeneous, clay-rich sedimentary rocks from a quarry near Kaiserslautern, Germany, deposited during the Permian. Figure 1 presents representative thin section micrographs of the Yellow Felser sandstone, captured under Both polarized and normal transmitted light using optical microscopy at magnifications of 10X (binoculars) and 4–50X (objective lenses). The principal minerals were identified and annotated in the micrographs according to standard petrographic criteria. Quantitative analysis of the mineralogical composition was further supported by X-ray diffraction (XRD) measurements conducted on powdered rock samples. The combined results from thin section petrography and XRD reveal that the rock comprises a mixture of grain minerals, including quartz, feldspar, and amphibole, with a matrix predominantly consisting of clay minerals such as illite and kaolinite, as well as chlorite and muscovite. Notably, the Yellow Felspar sample exhibits a high clay content, as evidenced by the abundance of clay minerals within the matrix. The relative volume proportions of these mineral groups, as determined from the analyses, are summarized in Table 1. The selection of the Yellow Felser sandstone was motivated by two key factors: its high porosity and substantial clay content, which maximize the hypothetical potential for hydrogen-brine-clay interactions, and its relative homogeneity with reproducible geomechanical behavior, which minimizes uncertainties due to heterogeneity.

Two groups of samples, differing in dimensions, were prepared from the same block of Yellow Felser sandstone. The first group comprised cylindrical samples with a diameter of 30 mm, which were later cut to a length of 60 mm– the standard size for rock mechanical tests. The second group consisted of disc-shaped samples, 30 mm in diameter and 6 mm thick, prepared for surface imaging purposes. The connected porosity of the samples prior to any exposure (brine or hydrogen) was measured using a helium pycnometer and found to be 21 ± 0.5%. To ensure reproducibility in subsequent tests, only samples with porosity within ±1% of the average were selected.

A synthetic brine was used, which consists of the following diluted concentrations: 16.2642 g/L of NaCl, 0.2948 g/L of KCl, 7.858 g/L of $CaCl_2\cdot$ $2\hbox {H}_2$O, and 1.816 g/L of $\hbox {MgCl}_2\cdot$ $6\hbox {H}_2$O, resulting in a total dissolved solids (TDS) concentration of 26.233 g/L. To expose rock samples to hydrogen, a Proserv high-pressure, high-temperature vessel was used, as shown in Fig. 2. This pressure cell is capable of pressurizing up to 680 bar and heating up to $177^{\circ }$C. A Memmert oven was used to elevate the temperature. Additionally, a pure (99%) hydrogen tank or capsule was connected to the cell to introduce and pressurize hydrogen inside.

**Table 1:** Mineralogical composition of Yellow Felser sandstone.

**Grain minerals**		**Matrix minerals**	
	**Volume %**		**Volume %**
Quartz	34	Illite	16
Feldspar	34	Kaolin	11
Epidote	2	Muscovite	3
**Total**	**70**	**Total**	**30**

### Exposure protocol

Two exposure methods were employed to investigate the effects of hydrogen/brine interactions with sandstone. Before exposure, the samples underwent a series of pre-exposure tests, including porosity measurements, ultrasonic P-wave measurements and SEM (scanning electron microscopy) surface imaging.

In the first method, samples were exposed only to brine, using high temperature-tolerant, sealed glass Bottles to ensure chemical stability and prevent contamination. They were fully saturated using a vacuum desiccator overnight at room temperature. Due to the high porosity of the samples, overnight saturation was sufficient to achieve full saturation. After full saturation, the samples were placed inside an oven cabin and kept there for six months. After placing the samples in the oven, the temperature was raised to 100°C. All exposures in this method were conducted at ambient pressure.

In the second method, samples were exposed to hydrogen-saturated brine. The samples were vacuum saturated with brine for one hour and then carefully placed into a high-pressure, high-temperature vessel, as shown in Fig. 2. Once positioned, the vessel was vacuumed again to remove any remaining air. Hydrogen gas was then introduced by opening the inlet valve, and the pressure was gradually increased to 150 bar. The inlet valve remained connected to the hydrogen cylinder to maintain pressure in case of leakage, reactions, and hydrogen dissolution. Simultaneously, the oven was turned on to raise the temperature to $100~^{\circ }$C (representative of deep reservoir). The samples were kept inside the vessel and exposed to hydrogen for six months. After six months of exposure, both groups of samples (hydrogen-saturated brine and brine-only) were removed and subjected to P-wave velocity measurements and SEM imaging. Following these measurements, samples from both groups were used for geomechanical testing. At no point were the samples dried; they were continuously kept saturated with the same synthetic brine used during the exposure phase to preserve pore fluid conditions and avoid any salt precipitation.

### Geomechanical testing apparatus and protocol

**Fig. 3:**
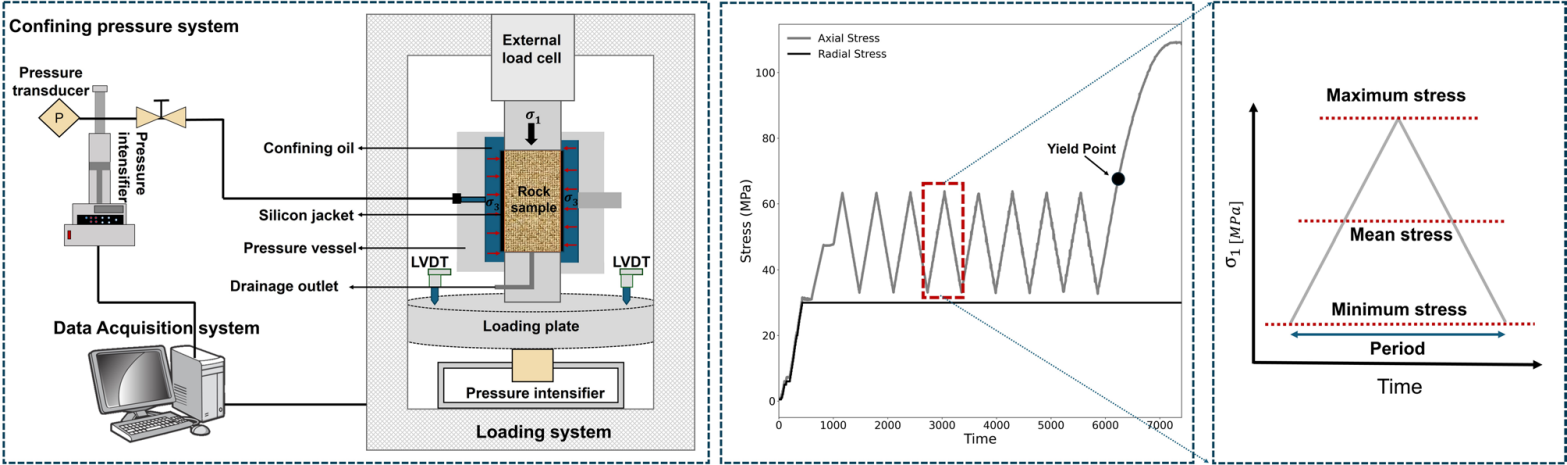
Schematic of the experimental setup: (**a**) pressure vessel in an oven at $100^{\circ }\hbox {C}$, (**b**) top view of the vessel, (**c**) prepared rock samples.

To conduct the triaxial cyclic tests, also referred to as the deviatoric cyclic test, a servo-controlled loading machine developed at TU Delft was used to apply axial stress ($\sigma _1$), as shown in Fig. 3. To apply confining pressure or horizontal stress, i.e., $\sigma _2 = \sigma _3$, an instrumented triaxial cell–shown in Fig. 3–was employed alongside the loading machine. The triaxial cell is equipped with a specialized silicon jacket that isolates the rock sample from the confining fluid. Vertical deformation of the rock sample is measured using two Linear Variable Displacement Transformers (LVDTs), as also depicted in Fig. 3.

After completing non-destructive measurements on both groups of exposed samples, the specimens were subjected to triaxial cyclic testing. To characterize the failure stages, particularly the brittle yield point (the onset of non-linear behavior), and the deformation behavior of the Yellow Felser sandstone, two initial monotonic deviatoric triaxial tests were performed at different confining pressures, continuing until macroscopic failure occurred. Three different confining pressures ($C_p$), i.e., 10, 20, and 30 MPa, were applied, representing various reservoir depths and enabling the construction of yield and failure envelopes for the two groups of exposed samples. To investigate the effect of cyclic loading conditions, specific frequency (*f*) and amplitude (*A*) values were selected such that all cycles remained within the linear elastic regime of the stress-strain response, considering the applied confining pressure. There were two main reasons for remaining within the elastic regime during testing. First, in real reservoir field conditions, it is recommended to maintain a safe stress state within the elastic range to avoid operational risks associated with exceeding the reservoir stress limits. Second, staying in the elastic regime allows to isolate and identify changes resulting primarily from geochemical effects, rather than those caused by stress-induced damage that occurs above the brittle yield point.

The amplitude and onset of cyclic stress were further adjusted according to the ratio of the yield point at each confining pressure, ensuring that cyclic loading remained entirely within the linear regime under all stress conditions. Specifically, at lower confining pressures, where the yield point is reduced, the stress amplitude was set accordingly to avoid exceeding the linear limit. In addition, the loading frequency was adjusted to maintain a consistent stress rate across all tests. As illustrated in Fig. 3, once the desired confining pressure (radial stress) was achieved, axial stress was increased in a deviatoric manner. Upon entering the linear regime, cyclic loading was performed, consisting of a total of eight cycles. After completing the cycles, axial stress was increased further until localized shear failure occurred. Table 2 summarizes the confining pressures, amplitudes, and frequencies used for the tested samples.

**Table 2:** Information about rock samples, exposure conditions (temperature *T*, pressure *P*, and duration), cyclic parameters (amplitude *A* and frequency *f*), stress conditions (mean stress of cycles $\sigma _{\text {mean}}$ and confining pressure $C_{p}$), final strength $\sigma _{f}$, total inelastic strain $\varepsilon _{1,\text {total}}^{\text {inelastic}}$, and porosity.

Sample	Exposure condition	$\varvec{\sigma _{\text {mean}}}$ [MPa]	A [MPa]	*f* [Hz]	$\varvec{\varepsilon _{1,\text {total}}^{\text {inelastic}}}$ [%]	$\varvec{\sigma _{\text {f}}}$ [MPa]	$\varvec{C_{p}}$ [MPa]	Porosity [%]
YF17	$\hbox {H}_2$/brine ($T = 100~^{\circ }$C, $P = 150$ bar, 6 months)	48	30	0.0016	0.068	111.29	30	21.10
YF19	$\hbox {H}_2$/brine ($T = 100~^{\circ }$C, $P = 150$ bar, 6 months)	48	30	0.0016	0.088	112.86	30	20.93
YF33	brine ($T = 100~^{\circ }$C, $P = 1$ atm, 6 months)	48	30	0.0016	0.071	113.77	30	21.09
YF15	brine ($T = 100~^{\circ }$C, $P = 1$ atm, 6 months)	48	30	0.0016	0.082	109.27	30	20.56
YF12	$\hbox {H}_2$/brine ($T = 100~^{\circ }$C, $P = 150$ bar, 6 months)	41	26	0.0019	0.031	88.34	20	20.21
YF16	brine ($T = 100~^{\circ }$C, $P = 1$ atm, 6 months)	41	26	0.0019	0.045	95.14	20	21.8
YF13	$\hbox {H}_2$/brine ($T = 100~^{\circ }$C, $P = 150$ bar, 6 months)	31	21	0.0023	0.082	67.73	10	20.76
YF2	$\hbox {H}_2$/brine ($T = 100~^{\circ }$C, $P = 150$ bar, 6 months)	31	21	0.0023	0.044	71.51	10	20.81
YF15	brine ($T = 100~^{\circ }$C, $P = 1$ atm, 6 months)	31	21	0.0023	0.067	69.35	10	21.2

## Results

### Geomechanical characterization

#### Mechanical parameters, Axial stress-strain, Elastic properties

**Fig. 4:**
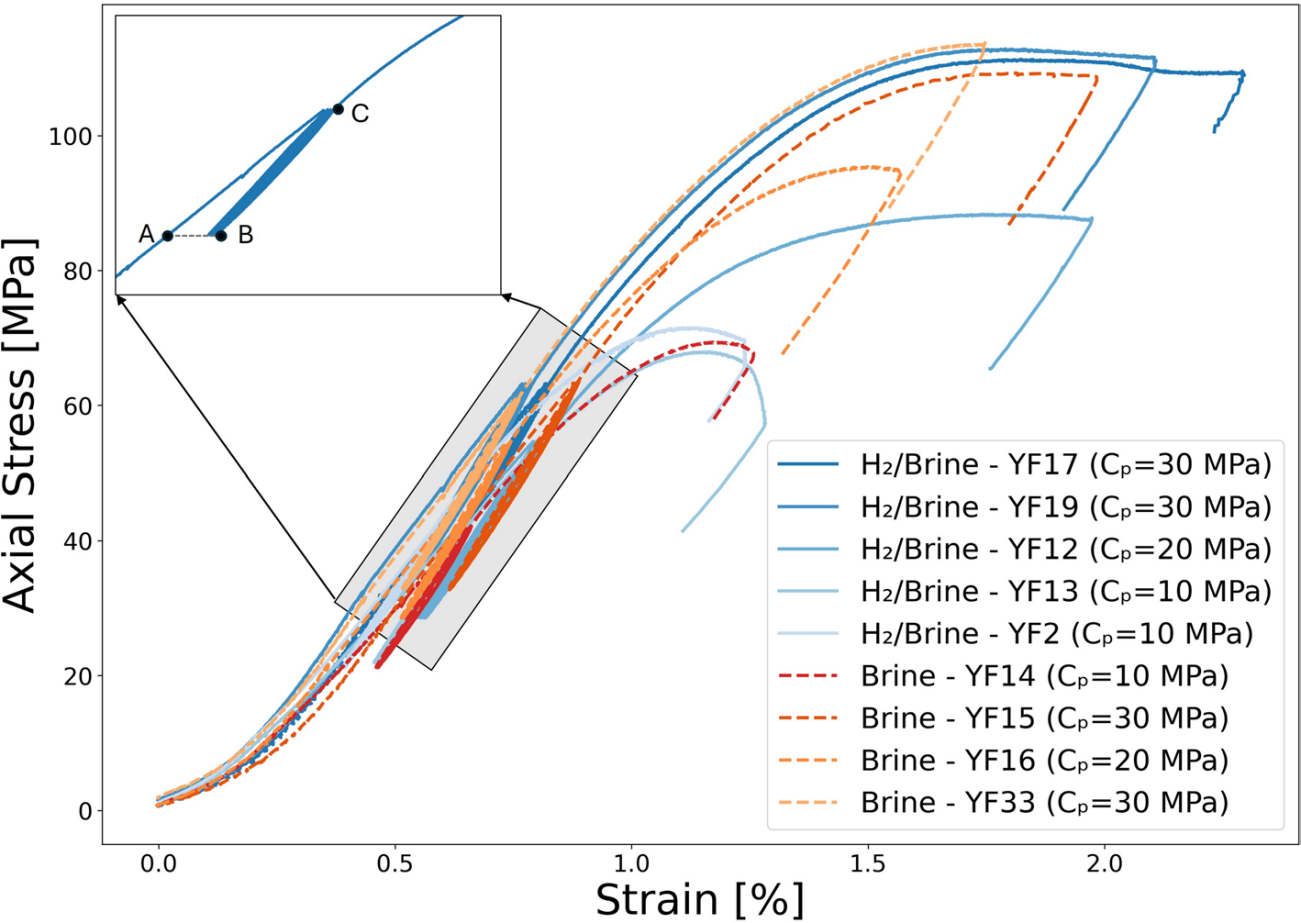
Stress-strain relationships of all experiments, comparing samples exposed to $\hbox {H}_2$/brine and brine-only conditions, each tested under varying confining pressures ($\hbox {C}_\textrm{p}$). The shaded rectangle indicates the general region where cycling occurred across tests. The inset shows a representative experiment (YF12) and marks: A–the start of cyclic loading, B–the end of the last cycle, and C–the subsequent monotonic phase in which the deviatoric stress is increased to failure. Because cycle start and finish vary among tests, markers are shown only in the inset rather than on every curve.

**Fig. 5:**
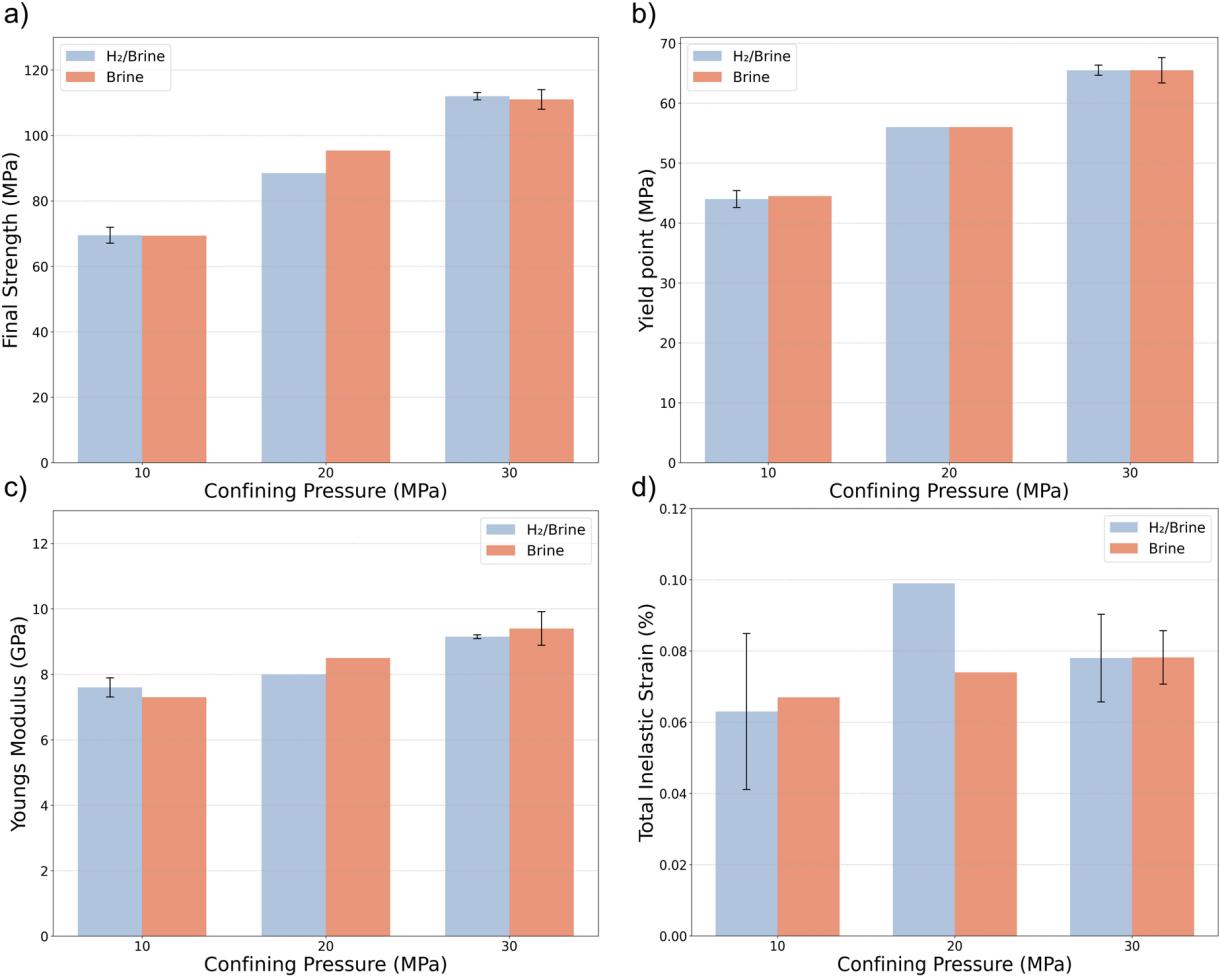
Comparison of different parameters for the two groups of samples: (**a**) final strength, (**b**) yield point, (**c**) Young’s modulus, and (**d**) total inelastic strain after eight cycles under different confining pressures. Note that the results for both low and high confining pressures represent averages from two repeated experiments. Where shown, error bars denote $\pm 1$ standard deviation from replicate tests at the same confining pressure; conditions tested only once are plotted without error bars.

Figure 4 illustrates the stress-strain relationships for samples exposed to hydrogen-saturated brine and brine-only conditions, each tested under varying confining pressures. In all experiments, the differential stress continues to increase after the cyclic loading phase, eventually reaching a final peak strength. Figure 4 shows that increasing the confining pressure results in a post-peak response characterized by a plateau in stress and ductile behavior, with no abrupt stress drop. In contrast, samples tested at the lowest confining pressure exhibit a more pronounced post-peak stress drop, indicative of more brittle behavior and localized shear failure. To better compare the effects of hydrogen exposure on the mechanical properties of the sandstone, various parameters were extracted from the stress-strain data, presented in Fig. 4. These parameters are the final strength, yield point, total inelastic strain, and Young’s modulus as presented in Fig. 5. As expected, both the yield point and maximum strength increase with confining pressure for both groups. However, no significant difference is observed between the samples exposed to hydrogen-saturated brine and those exposed to brine-only conditions, as shown in Fig. 5.

**Fig. 6:**
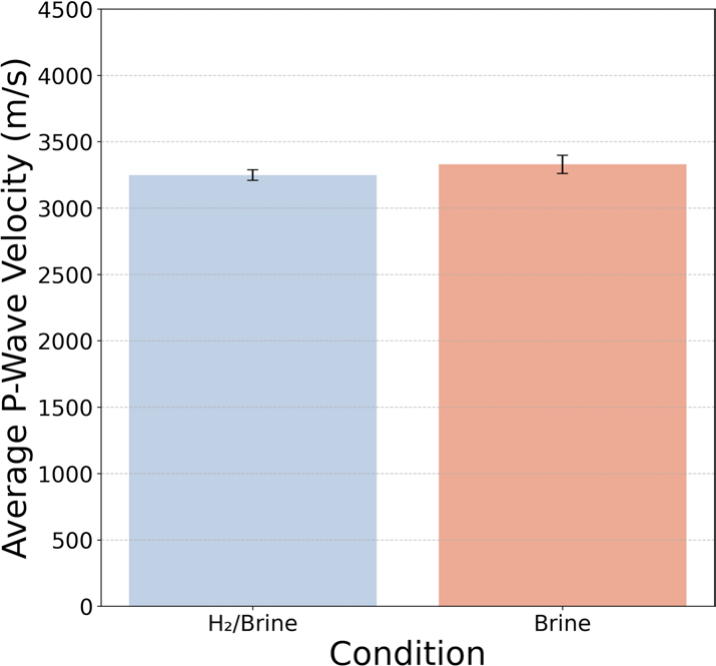
Comparison of average P-wave velocity (with standard deviation) measured in brine-saturated sandstone samples before and after exposure to hydrogen and brine.

Young’s modulus (estimated before the start of cyclic loading) is presented in Fig. 5c. Similar to the final strength and yield point, as shown in Fig. 5a and b, Young’s modulus increases with confining pressure, with no significant difference observed between the two groups of samples. At medium and high confining pressures, the Young’s modulus decreased by an average of approximately 3% following exposure. Figure 8 shows the evolution of Young’s modulus over the eight loading cycles, under different confining pressures. In all cases, the largest increase in Young’s modulus occurs between the first and second cycles, after which it stabilizes with only minor variations. This initial increase may reflect early closure of microcracks as a result of compaction. No significant differences are observed between the hydrogen-saturated brine and brine-only exposed samples in terms of modulus values or trends over the cycles.

Moreover, the total inelastic strain was determined by calculating the difference between the strain at the beginning of the cyclic loading and the strain at the end of the final unloading cycle, both measured at the minimum axial stress of the cycles and under consistent confining pressure. This method ensures a consistent reference point for assessing inelastic strain. The resulting inelastic strain can reflect both instantaneous inelastic deformation and time-dependent behaviors of the rock such as viscoelastic effects^10^. The values of total inelastic strain obtained for each confining pressure are presented in Fig. 5d. Total inelastic strain remains fairly similar for Both groups at low and high confining pressures; however, at medium confining pressure, the sample exposed to hydrogen-saturated brine exhibits 30% higher inelastic strain compared to the sample exposed to brine only. This difference may also be influenced by the slightly higher porosity of the sample which was exposed to hydrogen-saturated brine. Indeed, as shown in Table 2, the porosity of YF12 is approximately 1.3% greater than that of YF16. Interestingly, there is no significant increase in total inelastic strain with increasing confining pressure, indicating that most of the irreversible deformation occurs even at lower stress levels.

**Fig. 7:**
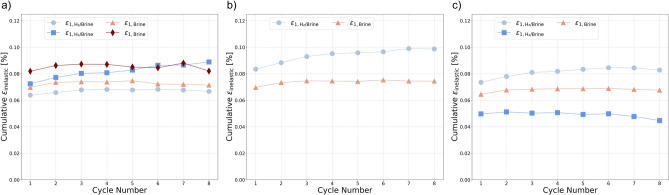
Evolution of inelastic strain versus the number of cycles: (**a**) experiment at high confining pressure (${C_{p}} = 30$ MPa), (**b**) medium confining pressure (${C_{p}} = 20$ MPa), and (**c**) low confining pressure (${C_{p}} = 10$ MPa).

Figure 7 shows the evolution of inelastic strain with the number of cycles under different confining pressures. The main observation, regardless of exposure condition, is that the first cycle accounts for the largest portion of inelastic strain. In the subsequent cycles, the rate of inelastic strain accumulation decreases significantly, and in some experiments, no further increase in inelastic strain is observed. A minor trend is observed with confining pressure: higher confinement generally leads to slightly larger induced cumulative inelastic strain. At medium confining pressure, the hydrogen-saturated brine exposed samples show higher cumulative inelastic strain compared to the brine-only exposed samples, and the inelastic strain continues to build up toward the final cycle. However, at low and high confining pressures, the cumulative inelastic strain of the hydrogen-saturated brine and brine-only samples is comparable when averaged across all cycles, indicating no clear enhancement or suppression of inelastic strain due to hydrogen exposure under these conditions. These observations suggest that the influence of hydrogen exposure is negligible on induced inelastic strain at different stress paths.

**Fig. 8:**
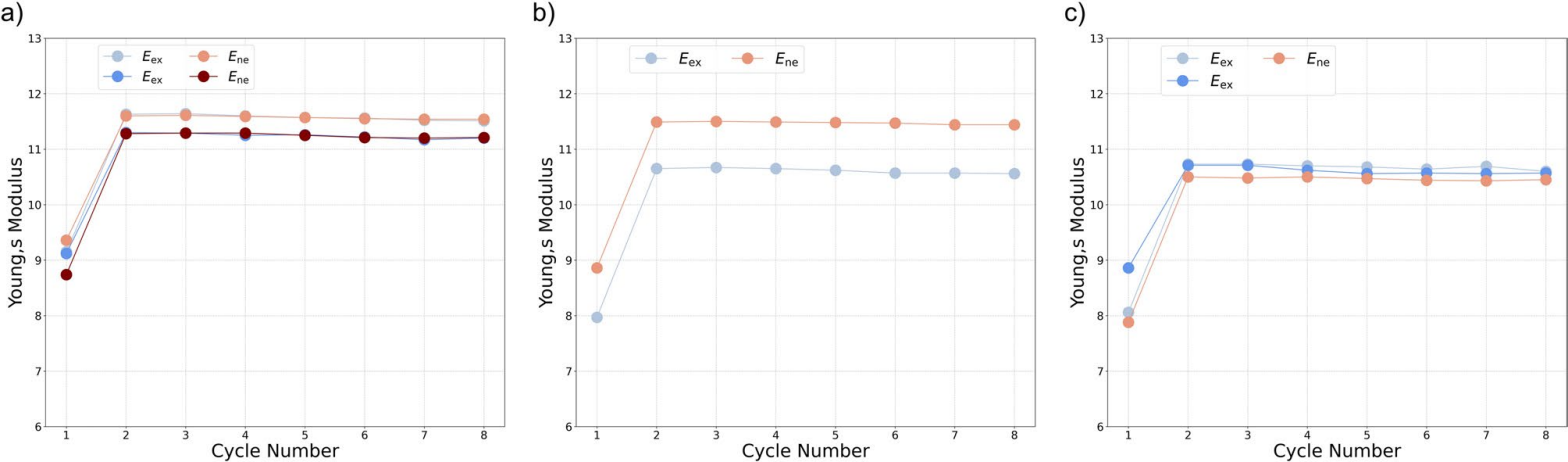
Evolution of Young’s Modulus versus the number of cycles: (**a**) experiment at high confining pressure (${C_{p}} = 30$ MPa), (**b**) medium confining pressure (${C_{p}} = 20$ MPa), and (**c**) low confining pressure (${C_{p}} = 10$ MPa).

As mentioned in the methodology section, ultrasonic measurements were conducted to determine the P-wave velocity before and after exposure on brine-saturated samples. Changes in ultrasonic properties can serve as indicators of alterations in the microstructure of the rock matrix^30^. The results are presented in Figure 6. The average calculated P-wave velocity exhibits an approximate 3% reduction following exposure to the hydrogen–brine system, indicating only a minor impact of the exposure on the acoustic properties of the rock.

#### Failure envelop

**Fig. 9:**
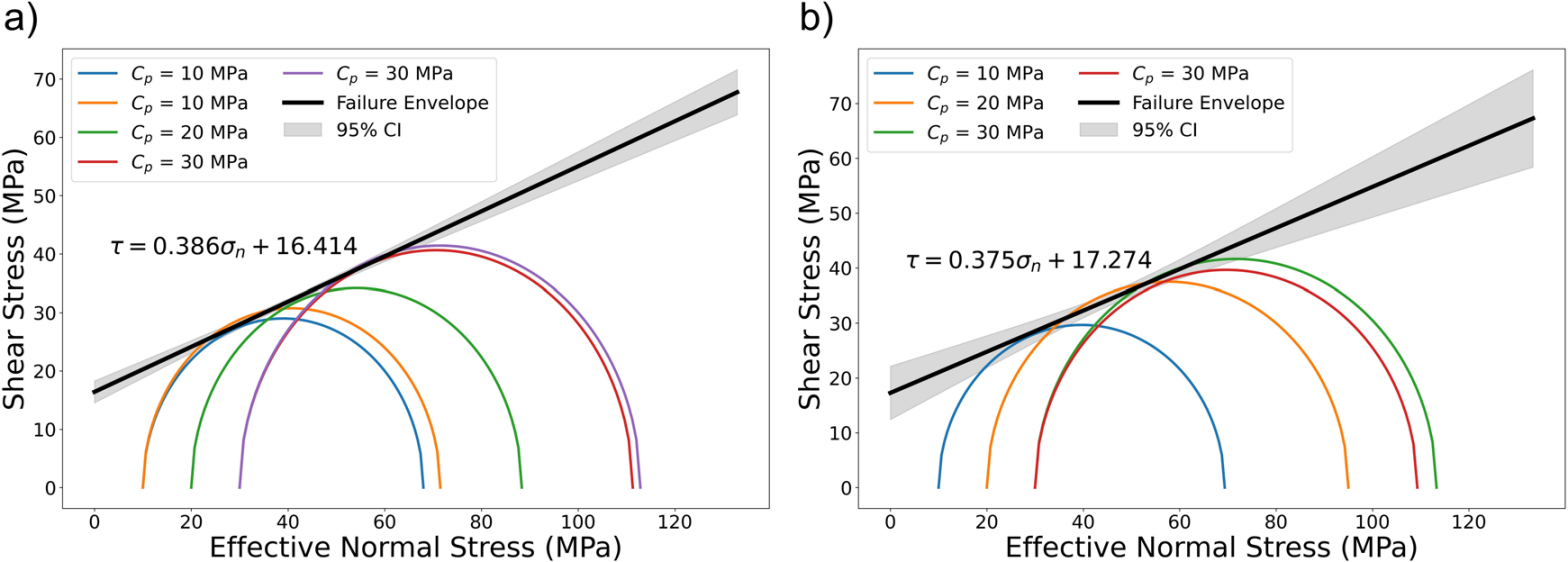
Failure envelop (Mohr-Coulomb) established in shear stress versus effective normal stress: (**a**) samples exposed to $\hbox {H}_2$/brine and (**b**) samples exposed to brine-only. A shaded 95% confidence band around each fitted envelope represents the confidence interval for the mean Mohr–Coulomb relation.

**Fig. 10:**
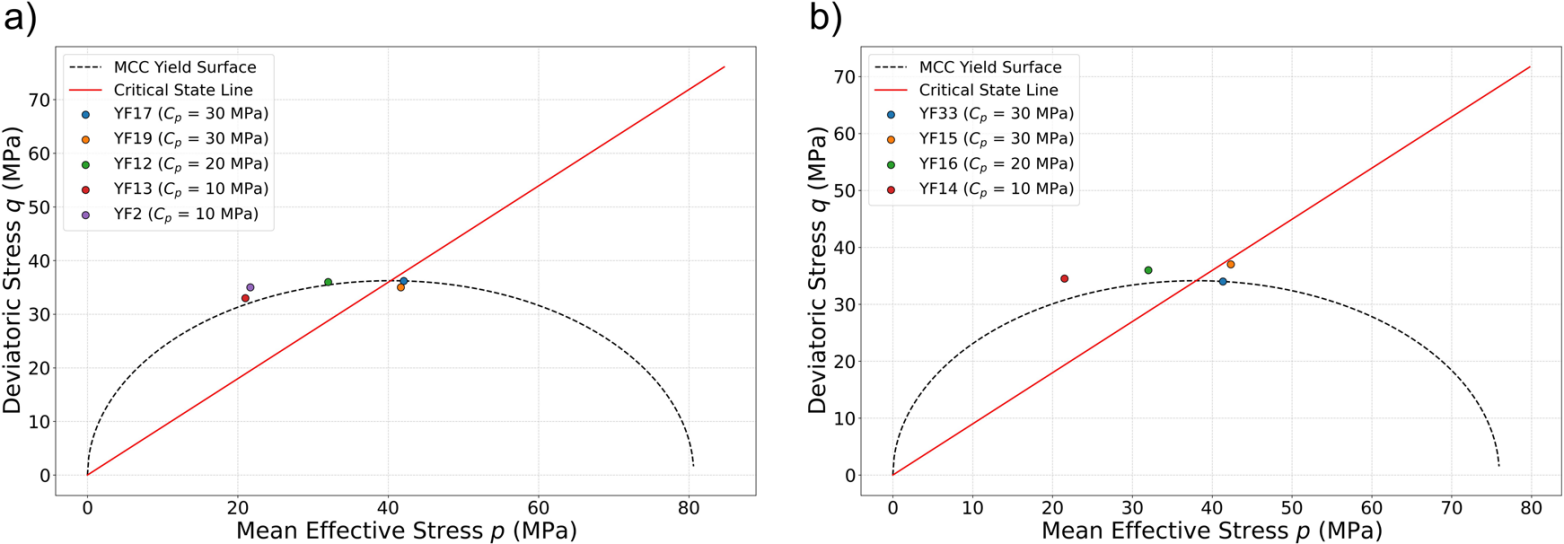
Evolution of yield points under different confining pressure: (**a**) samples exposed to $\hbox {H}_2$/brine and (**b**) samples exposed to brine-only.

Figure 9 presents the Mohr-Coulomb failure envelopes for samples exposed to hydrogen-saturated brine (a) and brine-only (b), plotted as shear stress versus effective normal stress. Although brittle shear faulting is not observed at medium and high confining pressures, it does occur under low confining pressure. This observation is supported by post-test visual inspection, which reveals a clear fault plane in the low-confinement samples, while no localized shear failure is observed in the samples tested at higher confinements. Despite this, the maximum deviatoric stress from each test was used to construct the failure envelopes in order to estimate the internal friction coefficient and cohesion for both groups of samples. The friction coefficients (slopes) are 0.386 for hydrogen-saturated brine and 0.375 for brine-only exposed samples, while the cohesion intercepts are 16.4 MPa and 17.3 MPa, respectively. This is shown in Fig. 9. These values indicate only minor differences exist between the two exposure conditions. Overall, the failure behavior remains consistent across both sets of samples within experimental variability, with no substantial change in frictional strength (less than 3%) or cohesion due to hydrogen exposure. The Modified Cam-Clay (MCC) model incorporates a yield surface to distinguish between elastic and inelastic behavior, as well as a critical state line (CSL) that defines material failure and the subsequent hardening or softening^10,31^. Figure 10 shows the yield envelope for both groups of samples in the space of deviatoric stress (*q*) versus effective mean stress (*p*). Both groups of samples exhibit similar yielding behavior, and for both, increasing the confining pressure results in a transition toward more ductile behavior as the samples pass the critical state line.

### Scanning electron microscopy (SEM)

**Fig. 11:**
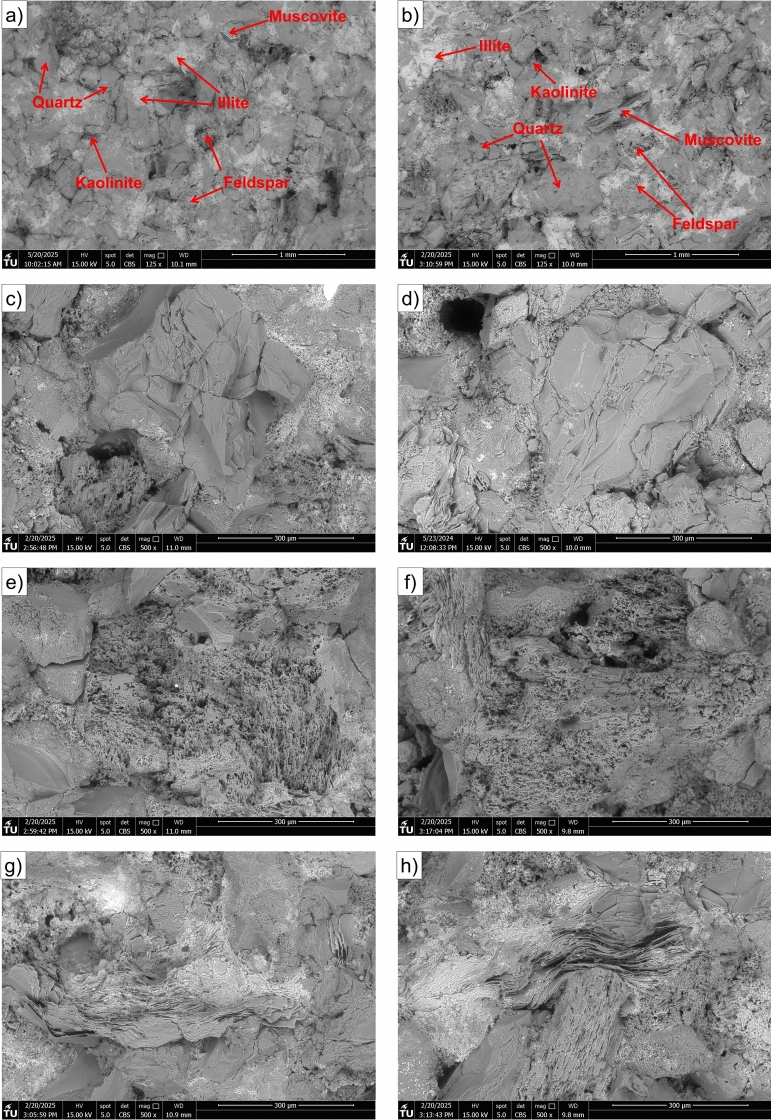
Scanning Electron Microscope (SEM) images of Yellow Felser sandstone samples exposed to hydrogen/brine solution (left column: **a**, **c**, **e**, **g**) and brine only (right column: **b**, **d**, **f**, **h**), taken at various magnifications to highlight different mineralogical components. The top row (**a**-**b**) provides low-magnification overviews showing the general texture and grain arrangement. The second row (**c**-**d**) focuses on quartz-rich regions, while the third row (**e**-**f**) highlights feldspar grains, and the bottom row (**g**-**h**) presents clay-rich zones.

The SEM analysis provides comprehensive insights into the microstructural characteristics of Yellow Felser sandstone samples following exposure to hydrogen-saturated brine and brine-only. The SEM images presented in Fig. 11 show representative microstructures of Yellow Felser sandstone samples subjected to two different fluid exposures: hydrogen-saturated brine (left column: a, c, e, g) and brine-only (right column: b, d, f, h). These images cover various magnification levels to highlight the overall texture (top row), quartz grains (second row), feldspar phases (third row), and clay-rich zones (bottom row). It is important to note that the images were taken from different locations within the respective samples, and not from identical spots, which may naturally lead to local heterogeneities in their microstructure. Across all rows, the overall microstructural characteristics appear comparable between the two groups. In the overview images, as shown in Fig. 11a and b, both samples display a similar compact sandstone texture with angular grains and intergranular porosity. The quartz grains, as shown in Fig. 11c and d, exhibit conchoidal fracture surfaces and well-preserved boundaries, with no distinguishable differences in surface alteration or secondary precipitation features. Similarly, the feldspar-rich regions, as illustrated in Fig. 11e and f, show intact grain boundaries and minimal signs of dissolution or etching in both conditions, suggesting that fluid-rock interaction under the given exposure conditions did not induce observable microstructural changes. The clay fabrics, shown in Fig. 11g and h, in both samples remain relatively compact and aligned, with no clear indication of swelling, deflocculation, or disaggregation that could be attributed to the presence of hydrogen.

**Fig. 12:**
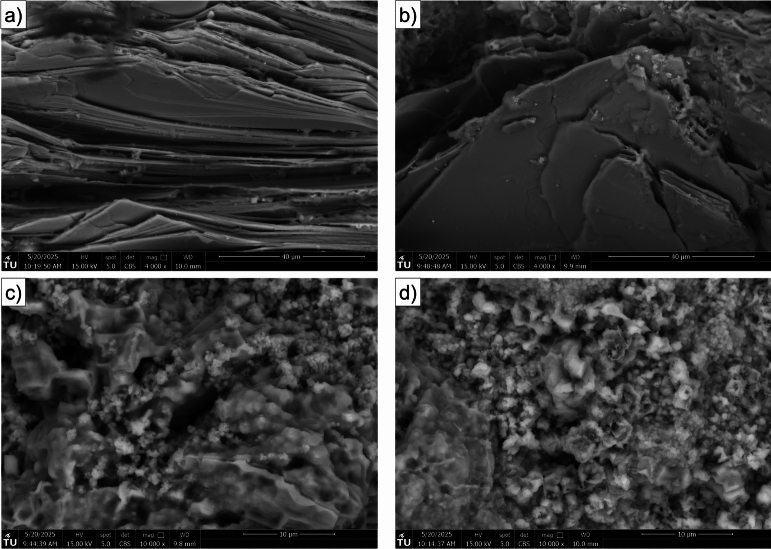
high-magnification SEM images of muscovite (**a**, **b**) and illite (**c**, **d**) minerals from samples exposed to hydrogen and brine (**a**, **c**) and brine only (**b**, **d**).

Figure 12 presents high-magnification SEM images of muscovite (a, b) and illite (c, d) minerals from samples exposed to hydrogen-saturated brine (a, c) and brine only (b, d). The images reveal well-preserved crystal morphology and surface textures in both minerals, with no evidence of dissolution, surface alteration, or structural degradation attributable to hydrogen exposure. Comparison between the two exposure conditions shows no meaningful difference in the microstructure of either muscovite or illite minerals.

**Fig. 13:**
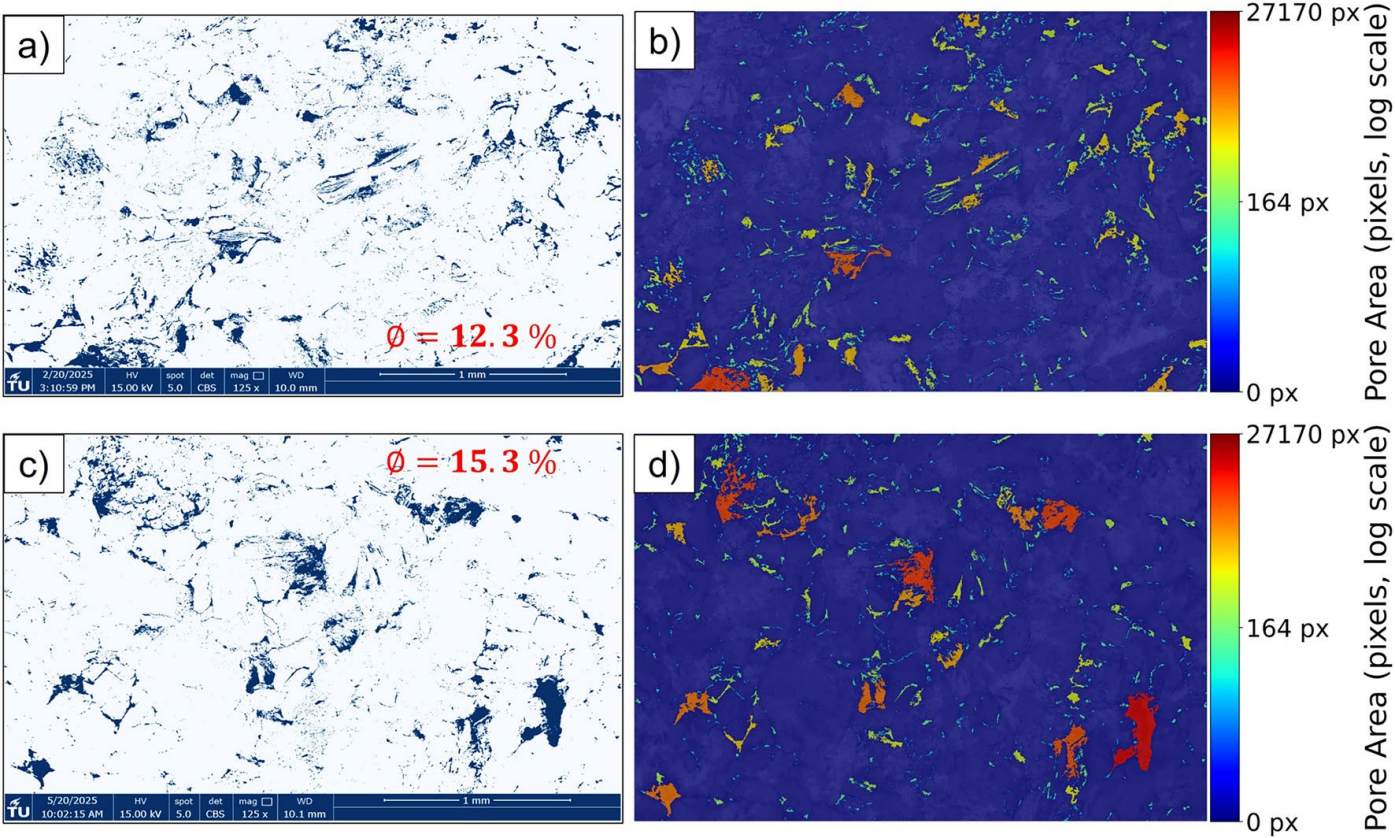
SEM image analysis results for sandstone samples exposed to hydrogen/brine (top row: a-b) and brine only (bottom row: c-d). (**a**, **c**) Pore segmentation maps highlighting pore spaces in blue. (**b**, **d**) Pore area distribution maps, where color indicates the size of individual pores on a logarithmic scale. The same color scale is used for both conditions to allow direct comparison. Regions were selected to be representative of the sample, ensuring that all major mineral phases were included within the field of view.

**Fig. 14:**
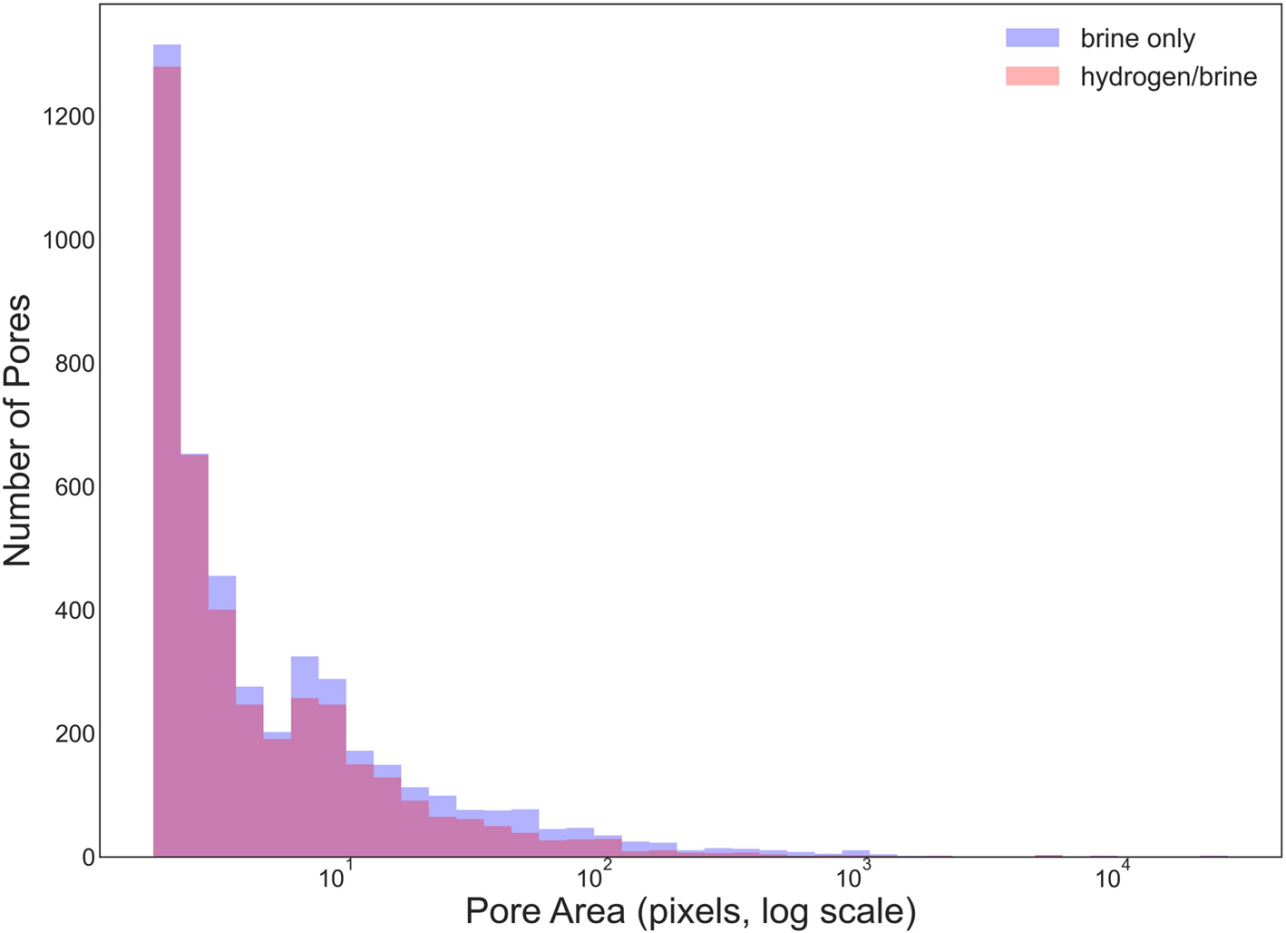
Pore area distributions for sandstone samples exposed to hydrogen/brine and brine only, showing similar microstructural characteristics for both conditions.

To complement the qualitative observations, both qualitative and quantitative image analyses were carried out to further explore the effects of hydrogen-saturated brine exposure on sandstone samples. Figure 13 provides a qualitative comparison, showing pore segmentation maps and pore area distribution for both sample groups. Fig. 13a and c show pore segmentation maps obtained by applying global Otsu thresholding to normalized SEM images^32^. SEM micrographs were normalized and converted to 8-bit grayscale. Pores were segmented with a global Otsu threshold; pixels with intensity $I(x,y) < T$ were labeled as pore, where $I$ is the grayscale intensity and $T$ is the threshold computed from the image histogram. Porosity was calculated as the ratio of pore pixels to total image pixels, yielding 12.3% ($\hbox {H}_2$/brine) and 15.3% (brine only). To evaluate segmentation robustness, the threshold was systematically varied by $\pm 5\%$ and $\pm 10\%$, which confirmed that porosity estimates remained stable within these ranges. It is clear that the porosity values fall within a comparable range for both hydrogen-saturated brine and brine-only conditions, although minor differences are present and may simply reflect the natural heterogeneity of the rock associated with the particular regions selected for the SEM analysis. For a more quantitative assessment, Fig. 14 presents pore area distribution for each group. Pore size distributions were obtained from segmented SEM images by measuring connected pore areas and excluding single-pixel artifacts to reduce noise. The pore areas, extracted from the labeled binary masks, were then plotted as frequency histograms using logarithmically spaced bins to capture both small and large pores. Pore size distribution follow similar trends for hydrogen-saturated brine and brine-only samples, with most pores in the small-size range and a comparable range of larger pores. Together, these qualitative and quantitative image analyses suggest that any small differences observed between the two exposure conditions are likely attributable to sample variability and selection of the analyzed regions.

## Discussion

### Potential geochemical reactions and Swelling

Mineral precipitation and dissolution through abiotic reactions in the presence of hydrogen theoretically have the potential to alter porosity and mineral morphology^11,15^, where estimated magnitudes are minimal or remain poorly constrained. Such alterations are more commonly expected in clay-rich rocks than in quartz- and feldspar-rich rocks, particularly those containing iron-bearing clays. These alterations, in turn, may influence rock strength, elastic properties, and thus the overall mechanical integrity of the reservoir. However, even after the relatively long exposure in our experiments compared to those experiments done to date, our results show that there are no significant changes in yield point, final strength, inelastic strain, and Young’s modulus of clay-rich sandstone rocks, before and after hydrogen solution exposure. Furthermore, the comparison of internal friction coefficient and cohesion before and after exposure indicates less than a 3% difference, which falls within the range of experimental uncertainty and natural sample variability. P-wave velocity measurements also show a minor reduction of approximately 3% following exposure. Even these minimal differences may reflect, at least in part, the disparity in exposure pressures ($\approx$1 atm for brine-only versus 150 bar for $\hbox {H}_2$-brine) rather than hydrogen-specific reactions. Together, these observations suggest that little to no abiotic reaction occurs during the six-month exposure period. This conclusion is supported by the limited potential for chemical reactions between existing minerals and the hydrogen-saturated brine, as well as the minimal swelling behavior of clay minerals under the tested conditions^11,15,33^.

The main grain minerals are quartz and feldspar, both of which exhibit no surface alteration based on SEM imaging, as shown in Fig. 11a–f. Given the absence of reported reactions with these minerals in the literature^13,18,19^ and the lack of morphological changes observed in our analysis, it can be concluded that these minerals are unlikely to undergo any significant reactions under the tested conditions, which are in the representative pressure and temperature range for large scale subsurface H2 storage in depleted hydrocarbon reservoirs^34^. Within the rock matrix of the Yellow Felser sandstone, illite and kaolinite are present. SEM images of the clay phases before and after hydrogen solution exposure reveal no signs of deflocculation, disaggregation, or surface alteration, as shown in Fig. 11g and h and Fig. 12c and d. This observation is consistent with previous studies showing that non-iron-bearing clays, such as illite and kaolinite, exhibit low reactivity with hydrogen under subsurface-relevant conditions. Hassanpouryouzband et al.^13^ conducted batch experiments in which samples were exposed to hydrogen under subsurface-relevant conditions. The tested sandstones contained notable amounts of clay minerals, including illite (up to 2.65 wt%), kaolinite (up to 3.26 wt%), and muscovite (up to 2.71 wt%, combining both muscovite entries). Despite the presence of these Fe-bearing and phyllosilicate phases, no measurable changes in pore fluid chemistry were observed following the experiments, indicating minimal or no reactivity of hydrogen with these mineral assemblages under the tested conditions. Hydrogen was found to strongly alter framboidal pyrite through reduction, dissolution, and pyrrhotite formation, while showing no detectable interaction with minerals such as clays, quartz, or calcite at low temperatures^35^. This is consistent with findings by Didier et al.^36^, who reported negligible Fe(III) reduction in clays below $350\,^{\circ }$C, and noted their stability under more typical underground hydrogen storage conditions ($\sim 90\,^{\circ }$C). Iron-bearing clay minerals are likely to interact with hydrogen. While ${\hbox {Fe}^{3+}}$ is generally not reduced to ${\hbox {Fe}^{2+}}$ under typical UHS conditions, hydrogen adsorption into the clay structure can still occur^36^. Dabbaghi et al.^27^ observed that specimens exposed to hydrogen exhibited a 24-41% reduction in peak strength compared to brine-only samples, which XRD analysis linked to a decrease in dolomite content. This highlights that dolomite, unlike quartz, feldspar, and non-Fe-bearing clays, is particularly prone to hydrogen-brine reactions, making it a critical mineral phase in evaluating reservoir integrity. Since our studied samples do not contain iron-bearing clay minerals, the likelihood of hydrogen-induced redox reactions, such as the reduction of Fe(III) to Fe(II), is expected to be minimal under the tested conditions.

Clay minerals differ in their swelling behavior upon exposure to water, which is largely governed by their structural type and chemical composition. 1:1 clay minerals, such as kaolinite, are generally non-swelling and chemically inert. In contrast, 2:1 clays exhibit more variable behavior. For example, montmorillonite can swell by up to 1500% due to significant interlayer water uptake, while illite remains non-swelling owing to strong potassium bonding in its interlayer spaces^37,38^. Masoudi et al.^33^ investigated hydrogen sorption capacity in various clay minerals and shale caprocks through characterization and hydrogen sorption tests on dried and non-dried samples. They found swelling clays (especially montmorillonite) had higher hydrogen uptake when dried, while non-dried samples showed reduced sorption due to water competition. They concluded that hydrogen sorption by clays in fully water-saturated conditions is minimal and unlikely to significantly impact hydrogen transport. Hydrogen adsorption on various clay minerals has been investigated using both experimental and simulation-based approaches under subsurface-relevant conditions^39–41^. Significantly higher adsorption capacities were observed for sepiolite and palygorskite, while illite, kaolinite, and chlorite exhibited minimal hydrogen uptake. The adsorption trend generally followed: sepiolite> montmorillonite> illite> kaolinite > chlorite. Among tested gases, CO_2_ showed the highest overall adsorption capacity. In our sandstone samples, the dominant clay minerals identified are illite (15%), kaolinite (10%), and muscovite (2%). These minerals are known to exhibit non-swelling or only minimal swelling behavior. As such, the mineralogical composition of the rock inherently limits the potential for swelling, even in the presence of water. Moreover, the experiments were conducted in the presence of brine, which further suppresses clay swelling. The high ionic strength of brine lowers the osmotic potential, thereby limiting water uptake into any expandable interlayers. This salinity effect is well documented to reduce swelling pressures and promote the stability of clay structures^42^. Additionally, brine likely competes with hydrogen for sorption sites and saturates surface functional groups, further hindering hydrogen access to the clay.

In porous reservoirs, hydrogen encounters three coexisting environments: a dry-out zone dominated by dry $\hbox {H}_2$, a mixed zone with residual brine, and a fully brine-saturated zone. The bulk of the reservoir lies in the latter two; thus, $\hbox {H}_2$-saturated brine experiments are most representative of the dominant geochemical setting, whereas pure-$\hbox {H}_2$ (dry) exposures reflect only localized near-wellbore conditions^18,43^. With brine present, reactivity depends on aqueous hydrogen produced by dissolution of $\hbox {H}_2$. The solubility of hydrogen in brine is dependent on pressure, temperature, and salinity, with dissolution decreasing as salinity increases due to the ionic strength of the brine^19,44^. The temperatures required to initiate hydrogen–fluid–mineral reactions are commonly lower than those in dry conditions^21,45^. Consistent with this, Cheng et al.^43^ reported that abiotic geochemical reactions do not occur in the dry-out zone at reservoir-relevant temperatures, while even in wet systems such reactions are limited. Working with Buntsandstein sandstones, they specifically showed that, unlike in hydrogen-brine solution, no detectable alteration of anhydrite occurs under dry conditions. In this study we exposed Yellow Felser sandstone to hydrogen-brine solution, which reflects the conditions in the brine-saturated and mixed zones. As these zones constitute the majority of the pore space in underground hydrogen storage reservoirs, our experimental design is representative of the dominant reservoir environment. Taken together, the results of this study indicate that no sign of mineral reactions or swelling occurred in the sandstone samples after prolonged hydrogen exposure under subsurface-relevant conditions. The absence of changes in geomechanical properties and SEM-observed mineral morphology suggests that the dominant clay minerals–illite, kaolinite, and muscovite–remained stable throughout the experiments. These findings are applicable to clay-rich sandstones which are quartz-feldspar dominated and which contain clay assemblages dominated by non-swelling, Fe-poor phyllosilicates (illite, kaolinite, muscovite) under brine-saturated, subsurface-relevant pressure-temperature-salinity conditions. They should not be generalized to sandstones containing abundant reactive carbonates and/or sulfides (e.g., calcite, dolomite, pyrite) and/or to systems rich in swelling clays (e.g., smectite, montmorillonite), nor to dry or partially saturated regimes, where reaction pathways and chemo-mechanical responses may differ substantially.

### Cyclic stress influence

As shown in Fig. 5c and d, no significant change is observed in either total inelastic strain or Young’s modulus following exposure to the hydrogen-saturated brine, compared to the brine-only exposure. A key observation across both experimental groups is that, under cyclic loading within the linear elastic regime, the majority of inelastic strain occurs during the initial loading cycles. Subsequent cycles do not result in further accumulation of inelastic strain, indicating a mechanically stable response over time, as shown in Fig. 7. The inelastic strain observed in this regime is primarily attributed to the compaction of clay coatings, the closure of pre-existing microcracks, and negligible new crack formation^46,47^. While physical changes such as mineral precipitation, pore structure alteration, or clay swelling–potentially induced by hydrogen-rock interactions–could affect inelastic deformation^9^, these processes are unlikely to occur under the tested conditions. This is due to the low reactivity and swelling potential of the existing mineral assemblage in the Yellow Felser sandstone^15^. Consequently, the absence of progressive inelastic strain is consistent with the anticipated mechanical stability following hydrogen exposure.

**Fig. 15:**
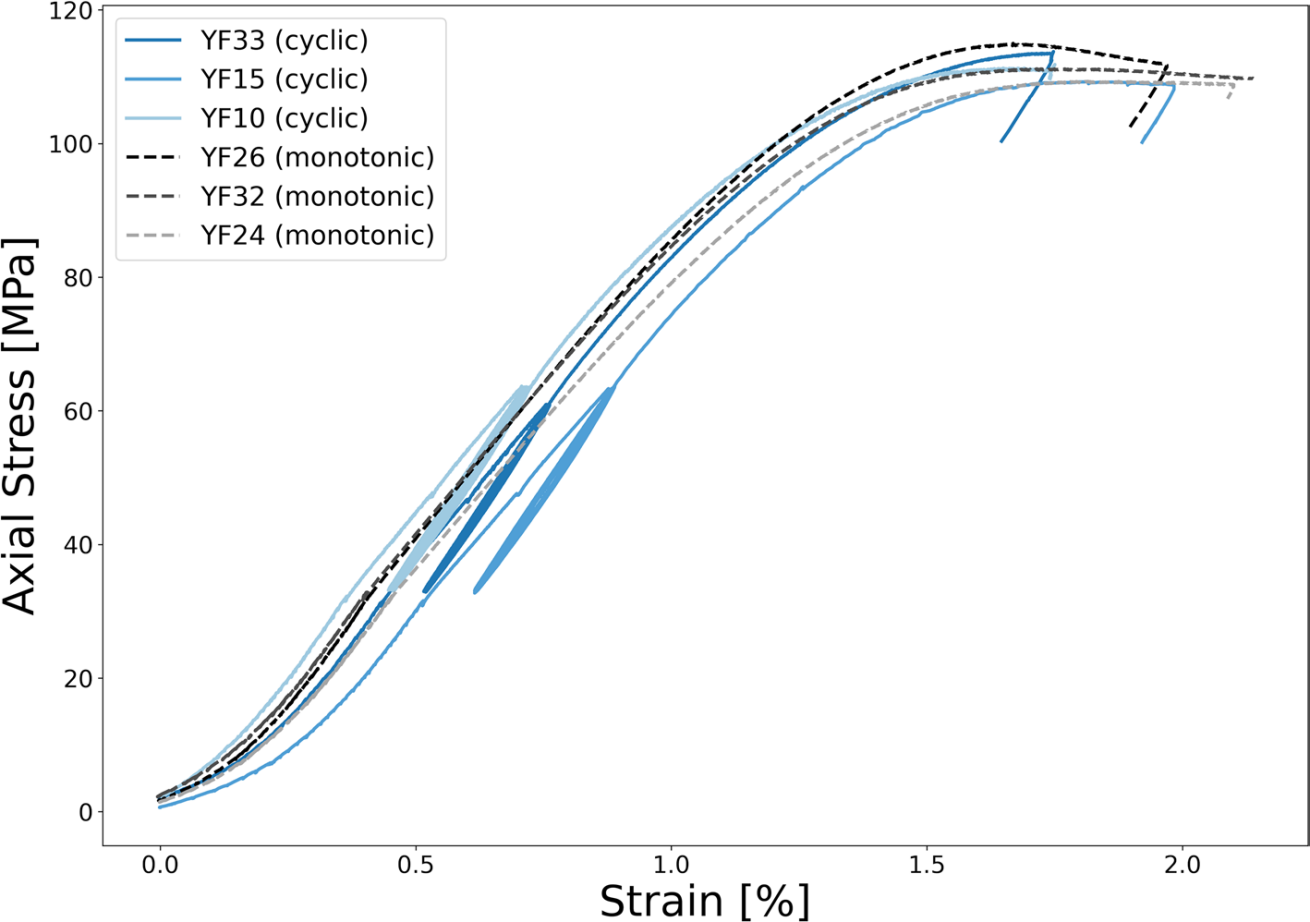
Comparison of stress-strain behavior between samples tested under monotonic loading to failure and those subjected to cyclic loading within the elastic range.

In addition to investigating the effects of hydrogen solution exposure, monotonic loading tests were also conducted to assess the influence of cyclic stress on the mechanical behavior of Yellow Felser sandstone. Figure 15 presents a comparison of stress-strain responses between samples subjected to monotonic loading until failure and those exposed to cyclic loading within the elastic regime, Both performed under identical confining pressure conditions of 30 MPa. Based on the extracted maximum axial stress from the stress-strain data, the average final strength of samples tested under monotonic loading was found to be 111.82 ± 2.90 MPa, while that of samples subjected to cyclic loading was 111.62 ± 2.26 MPa. This finding suggests that the application of cyclic stress conditions, even by inducing inelastic strain, does not affect the strength of yellow Felser sandstone. Naderloo et al.^10^ demonstrated that, when cyclic loading is applied within the linear elastic regime and below the brittle yield point, using constant-amplitude cycles on a quartz-rich sandstone, no additional inelastic strain accumulates beyond the initial cycle. This indicates that the primary inelastic response occurs early, with minimal progressive damage during subsequent cycles. Other studies have explored the effects of cyclic loading on final strength and damage mechanisms; however, variations in cycle amplitude (e.g., progressively increasing amplitudes), duration, frequency, and stress conditions (e.g., confined vs. unconfined) complicate direct comparisons^46,48–50^. Therefore, evaluating the impact of cyclic loading requires careful consideration of stress conditions, rock type, cycle amplitude, and loading frequency^51,52^. At higher stress levels and lower frequencies, time-dependent processes such as creep and stress corrosion may significantly influence damage evolution^10,53,54^. In the present study, the application of eight cycles under laboratory timescales did not affect the final strength of the rock, either before or after hydrogen solution exposure. We hypothesize that the inelastic strain observed during the first cycle is primarily due to the closure of pre-existing microcracks and the compaction of clay coatings–processes that do not significantly influence the overall mechanical behavior of the rock samples. To further investigate the effects of cyclic stress changes induced by hydrogen injection and withdrawal, in combination with abiotic reactions, future studies should move beyond cyclic stress testing under static exposure conditions and instead focus on active exposure scenarios that more closely replicate reservoir operations. Such approaches include implementing injection and production through controlled imbibition-drainage sequences, and coupling geomechanical testing with simultaneous hydrogen injection and depletion.

## Conclusion

This study investigated the geomechanical alterations in clay-rich sandstone (Yellow Felser) resulting from six months of exposure to a hydrogen-saturated brine system under static conditions (i.e., no hydrogen flow) at a pressure of 150 bar and a temperature of $100\, ^{\circ }\hbox {C}$. Triaxial cyclic loading tests were performed to assess changes in strength, elastic properties, inelastic strain, and the failure envelope. In addition, qualitative and quantitative analyses of SEM images were conducted on samples exposed either to brine alone or to the combined hydrogen-brine environment.

The results indicate that six months of hydrogen-brine exposure had no discernible effect on the failure envelope, elastic properties, inelastic strain, or acoustic characteristics of the Yellow Felser sandstone. Internal friction, P-wave velocity, and Young’s modulus each exhibited changes of approximately 3%, suggesting very minimal geomechanical alteration. Complementary qualitative and quantitative analyses using scanning electron microscopy (SEM) revealed negligible microstructural changes, further supporting the mechanical stability of the rock under the tested conditions.

When eight stress cycles were applied within the linear elastic regime, the majority of inelastic strain occurred during the first cycle, with no significant accumulation in subsequent cycles. Furthermore, comparison of stress-strain behavior between samples tested under monotonic loading to failure and those subjected to cyclic loading within the elastic range showed that cyclic loading, under these conditions, does not influence the mechanical behavior of Yellow Felser sandstone in terms of rock strength.

## Data Availability

The experimental data are openly available to the public through the 4TU.ResearchData repository at https://doi.org/10.4121/216f2aac-8783-4596-83d0-b6f4fae4a2a2.v1.
